# Addressing verapamil-sensitive idiopathic left ventricular tachycardia via catheter ablation targeting Purkinje potential on a false tendon: Rationale for using a high-resolution ablation catheter with distal-tip microelectrodes

**DOI:** 10.1016/j.hrcr.2025.05.002

**Published:** 2025-05-08

**Authors:** Kazuki Shimojo, Itsuro Morishima, Yasunori Kanzaki, Akihiko Nogami

**Affiliations:** 1 Department of Cardiology, Ogaki Municipal Hospital, Ogaki, Japan; 2 Arrhythmia Research Institute, Tokyo Heart Rhythm Hospital, Tokyo, Japan

**Keywords:** Idiopathic left ventricular tachycardia, Low irrigation flow, Microelectrodes, P1 potential, Purkinje potential, QDOT MICRO catheter

**Key Teaching Points**

• A high-resolution ablation catheter with 3 distal-tip microelectrodes that allow to record bipolar electrograms oriented perpendicular to those recorded by standard ablation electrodes enhanced the detection of Purkinje potentials.
• Thermocouples embedded in the electrode surface provide feedback on tissue heating, represented by a bullseye pattern, even under conditions of suboptimal contact forces.
• It is crucial to accurately identify intracardiac structures, especially the false tendon, using preoperative multimodality imaging and intraoperative intracardiac echocardiography, ensuring comprehensive mapping to detect relevant potentials.

## Introduction

Verapamil-sensitive idiopathic left ventricular tachycardia (ILVT) is a distinct subtype of ventricular tachycardia (VT) observed in young healthy individuals. It is characterized by monomorphic VT with a relatively narrow QRS complex, demonstrating a favorable response to verapamil. This condition is defined by specific electrophysiological features such as the diastolic Purkinje potential (P1) and a unique response to electrical stimulation.^1^^,^^2^ ILVT is associated with reentry involving Purkinje fibers and is sometimes linked to a false tendon within the VT circuit.^1^, ^2^, ^3^, ^4^ Catheter ablation can be challenging when the false tendon is the target.

This case report describes the successful treatment of a verapamil-sensitive ILVT using a QDOT MICRO catheter (Biosense Webster, Diamond Bar, CA). This catheter features additional microelectrodes on the ablation tip perpendicular to the standard ablation electrode. It uses a low irrigation flow at a 35-W radiofrequency (RF) application with a thermal titration function, effectively targeting P1 potentials and blocking the VT circuit on the false tendon.

## Case report

**Figure 1:**
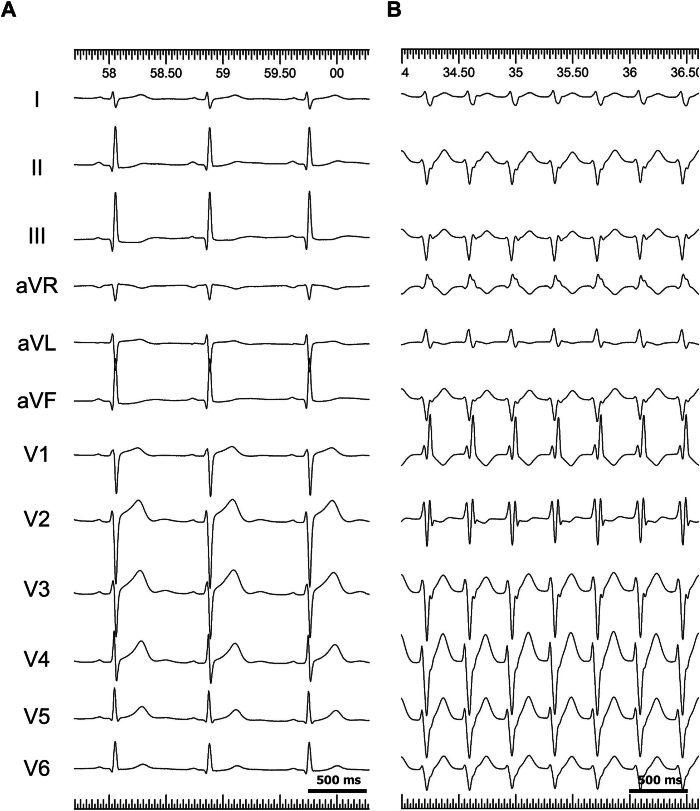
The 12-lead ECGs. **A:** Sinus rhythm. **B:** Clinical VT. Cycle length: 390 ms; right bundle branch block pattern; axis −94°; QRS duration: 116 ms. ECG = electrocardiogram; VT = ventricular tachycardia.

A 15-year-old man with a history of 2 unsuccessful catheter ablation sessions for verapamil-sensitive ILVT was referred to our institute for further treatment. Results of clinical examination and transthoracic echocardiography revealed no abnormalities other than a false tendon connecting the apex and the septum in the LV (Supplemental Movie 1). In addition, result of 12-lead electrocardiogram during sinus rhythm was unremarkable (Figure 1A).

After obtaining written informed consent, an electrophysiological study was performed in a lightly sedated, fasting state without antiarrhythmic drugs. At baseline, under sinus rhythm, the AH and HV intervals were 58 and 40 ms, respectively. The LV geometry was constructed using CARTOSOUND (Biosense Webster, Diamond Bar, CA), including details of the papillary muscles and false tendon. The clinical VT (cycle length: 390 ms, right bundle branch block pattern; axis: −94°; QRS duration: 116 ms; Figure 1B) was induced by spontaneous premature atrial contractions. Entrainment pacing from the atrium (coronary sinus ostium) and ventricle (RV outflow tract) demonstrated progressive fusion of the QRS complex.

**Figure 2:**
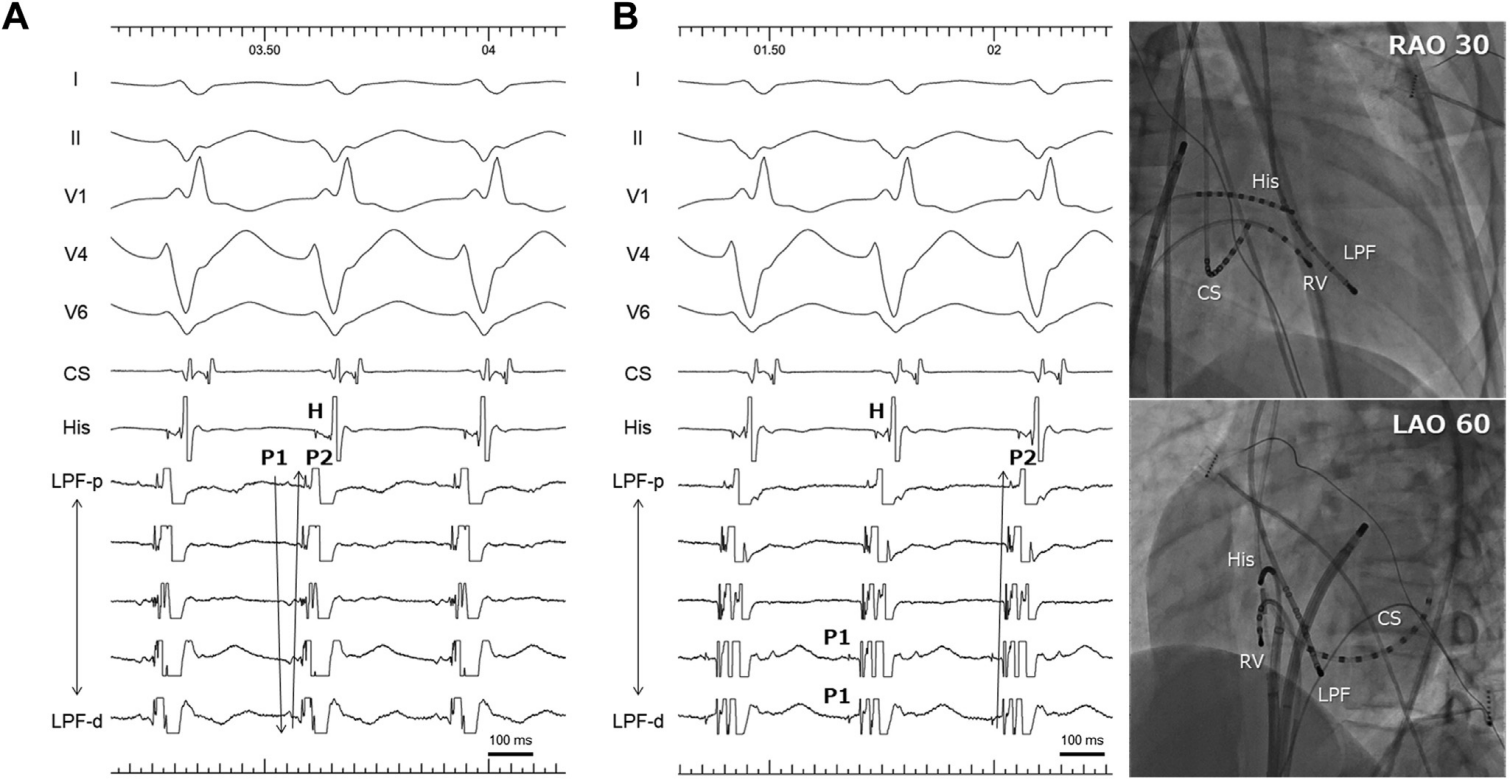
Mapping during VT. **A:** The DECANAV catheter is positioned on the left ventricular septum alongside the left posterior fascicle through a transaortic approach. Dull sequential P1 potentials preceding the QRS onset by 40 ms were observed from the base to the apex, accomplished by sharp retrograde P2 potentials. **B:** When DECANAV was adjusted from the septum to a slightly lateral position, sharp P1 potentials were detected. DECANAV identifies sharper P1 potentials because of its proximity to a false tendon near the left ventricular septum. LAO = left anterior oblique; RAO = right anterior oblique; VT = ventricular tachycardia.

**Figure 3:**
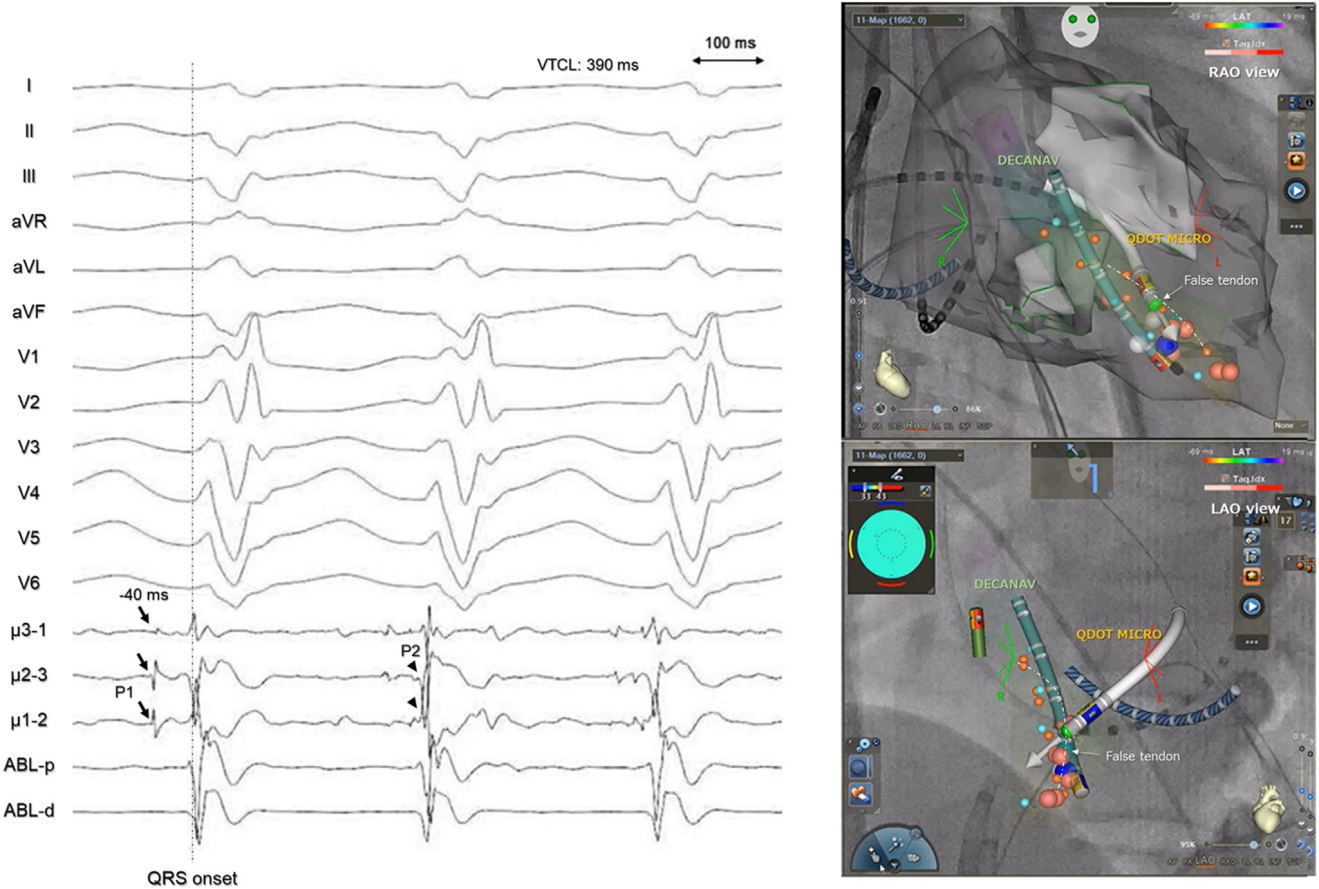
Successful ablation site. The microelectrodes detected the P1 potentials (arrows) exclusively, with no potential observed on the standard electrode of the ablation catheter. The image illustrates the QDOT MICRO catheter oriented perpendicular to the false tendon (P1 potential). The microelectrodes could detect P1 potentials using the transseptal approach, whereas conventional electrodes could not. The first RF application site elicited a response that terminated VT. LAO = left anterior oblique; RAO = right anterior oblique; Red tags = radiofrequency energy application sites through the transseptal approach; VT = ventricular tachycardia.

**Supplementary Figure S1:**
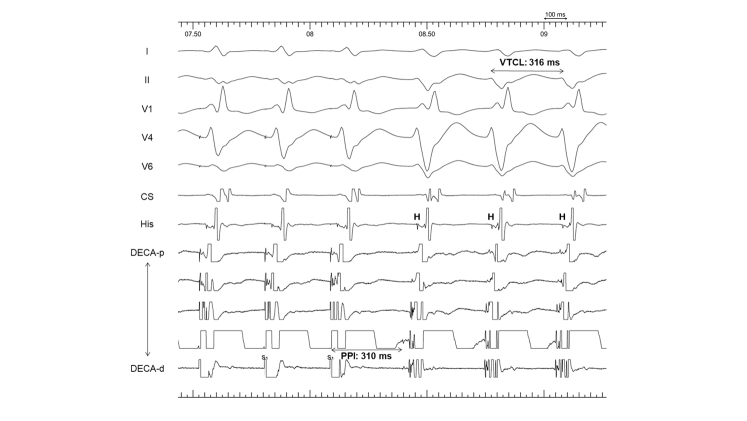
Entrainment pacing from the P1 site Ventricular entrainment pacing for VT performed from the distal electrode of the DECANAV catheter at the site detecting P1 potentials showed minimal fusion and a post-pacing interval that matched the tachycardia cycle length.

Using a DECANAV catheter positioned on the LV septum near the left posterior fascicle through a transaortic approach, dull sequential P1 potentials were observed preceding the QRS onset by 40 ms, extending from the base to the apex, along with sharp retrograde P2 potentials (Figure 2A). When DECANAV was slightly adjusted laterally, sharp P1 potentials were recorded (Figure 2B). Ventricular entrainment pacing from the distal electrodes of the DECANAV catheter at the site of P1 potential detection revealed minimal fusion and a post-pacing interval matching the tachycardia cycle length (Supplemental Figure 1). On the basis of these findings, a QDOT MICRO ablation catheter was advanced to the site where the P1 potential was recorded using a transseptal approach. Although P1 potentials were not visible in the bipolar recordings of the standard electrode pairs, they were clearly detected on the microelectrodes (Figure 3).

The RF energy applied at this site successfully terminated the VT; however, the VT remained inducible. Owing to the technical difficulty of catheter positioning, the ablation catheter was switched to a transaortic approach. Despite obtaining a weak (approximately 3–5 g) contact force (Supplemental Movie 2), 35-W temperature-controlled ablation at the site terminated the VT. During the RF application, tissue heating was confirmed using thermocouples on the electrode surface, even with a low contact force. Additional RF applications render VT no longer inducible. Intracardiac echocardiography revealed that the ablation site was on the false tendon, parallel to the LV septum (Supplemental Movie 3). The patient has remained free of VT recurrence for a year after the procedure.

## Discussion

Successful catheter ablation for ILVT is frequently performed on the LV mid-posterior septum.^1^^,^^2^ However, in this case, the P1 recording site was slightly away from the LV septum to the LV cavity, as demonstrated by mapping with the DECANAV catheter (Figure 2B, Supplemental Movie 3). Maintaining sufficient contact force at the successful site was technically challenging, and the contact vector indicated that the tip of the ablation catheter did not directly face the LV septum (Supplemental Movie 2). The successful ablation site corresponded to a false tendon bridging the septum and apex parallel to the LV septum (Supplemental Movie 3). Overall, the critical Purkinje fiber was most likely located on the false tendon, away from the septum, which may explain why previous ablation attempts were unsuccessful.

This case highlights the advantages of the QDOT MICRO ablation catheter than conventional irrigation ablation catheters. First, 3 additional microelectrodes placed on the catheter tip may have enhanced the likelihood of recording the P1 potential. Second, thermocouples on the electrode surface allowed for low irrigation flow during the 35-W RF application using a thermal titration function. Consequently, the thermocouples enabled the operator to confirm tissue heating even when the contact force was suboptimal.

The QDOT MICRO catheter features 3 additional microelectrodes on its tip, each with a small surface area of 0.086 mm^2^ and spaced 1.5 mm apart. These microelectrodes may improve the resolution of the Purkinje potential mapping during ILVT. Furthermore, the bipolar electrograms recorded by the microelectrodes are oriented perpendicular to those recorded by standard ablation electrodes. This design theoretically allows the detection of P1 potentials when the catheter is placed perpendicular to the P1 fibers, as in this case. Indeed, during transseptal mapping, the catheter was oriented perpendicular to the P1 fiber, which ran parallel to the septum. Although P1 potentials were not observed in the bipolar recording of the standard electrode pairs, they were clearly visible on the microelectrodes (Figure 3). The catheter enables the simultaneous recording of bipolar electrograms in 4 different directions (3 with microelectrodes and 1 with conventional electrodes), potentially increasing the likelihood of detecting P1 potentials. When using an ablation catheter without microelectrodes at the tip, an alternative strategy may be to target sites where stable P1 potentials are recorded by a high-resolution multispline mapping catheter. In our previous study, we reported a case in which such potentials were detected by the mapping catheter but not by the ablation catheter; nonetheless, ablation at that site led to successful treatment.^5^ Another possible therapeutic option is to anatomically target the insertion point of the false tendon on the septum, where catheter stability tends to be greater than on the tendon itself.^4^

The QDOT MICRO catheter also incorporates 6 thermocouples at its tip for precise local temperature measurements. Real-time feedback from these thermocouples allows dynamic power adjustment to maintain the target temperature and supports 35-W RF application with an irrigation flow rate as low as 4 mL/min. As the target Purkinje fibers are located superficially in the subendocardium, ablation with a low irrigation rate may be more effective than using a higher irrigation flow.^6^ The precise local temperature monitoring provides critical feedback for assessing whether an effective ablation lesion is being formed. In this case, an adequate contact force was difficult to achieve because the target Purkinje fiber was located on a small floating false tendon. However, the local temperature increased to 45°C (Supplemental Movie 2), confirming that the catheter contact was sufficient to create an ablation lesion on the surface of the false tendon.

## Conclusion

Considering these findings, the QDOT MICRO ablation catheter may be the optimal choice for the catheter ablation of ILVT, particularly in challenging cases.

## Disclosures

The authors have no conflicts of interest to disclose.

## Appendix Supplementary Data

Movie 1 Preoperative echocardiography Preoperative echocardiography indicates a false tendon (arrow) connecting the apex and septum in the LV.

Movie 2 Successful catheter ablation The QDOT MICRO catheter achieved a contact force of approximately 3–5 g, ensuring good perpendicular contact with the false tendon, as indicated by a bull eye thermometer. Despite applying only weak contact with a low irrigation flow to the floppy tissue of the false tendon, the procedure was successful. Green tag, successful ablation site; orange tags, P1-detected sites during ventricular tachycardia; light blue tags, P1-detected sites during sinus rhythm.

Movie 3 Intracardiac echocardiography Intracardiac echocardiography revealed that the ablation sites were near the false tendon, LV septum, and left posterior fascicle. Red tags, radiofrequency application sites.
