# Supplementary material for: Addressing verapamil-sensitive idiopathic left ventricular tachycardia via catheter ablation targeting Purkinje potential on a false tendon: Rationale for using a high-resolution ablation catheter with distal-tip microelectrodes
Source: HeartRhythm Case Rep. 2025 May 8;11(7):679–83. doi: 10.1016/j.hrcr.2025.05.002 (PMC12432856; doi:10.1016/j.hrcr.2025.05.002)
Supplement: Supplementary Material [file mmc4.docx]

**Supplementary Movie and Figure Legends**

**Movie 1.** Preoperative echocardiography

Preoperative echocardiography indicates a false tendon (arrow) connecting the apex and septum in the LV.

**Movie 2.** Successful catheter ablation

The QDOT MICRO catheter achieved a contact force of approximately 3–5 g, ensuring good perpendicular contact with the false tendon, as indicated by a bull eye thermometer. Despite applying only weak contact with a low irrigation flow to the floppy tissue of the false tendon, the procedure was successful.

Green tag, successful ablation site; orange tags, P1-detected sites during ventricular tachycardia; light blue tags, P1-detected sites during sinus rhythm.

**Movie 3.** Intracardiac echocardiography

Intracardiac echocardiography revealed that the ablation sites were near the false tendon, LV septum, and left posterior fascicle.

Red tags, radiofrequency application sites.

**Supplementary Figure S1.** Entrainment pacing from the P1 site

Ventricular entrainment pacing for VT performed from the distal electrode of the DECANAV catheter at the site detecting P1 potentials showed minimal fusion and a post-pacing interval that matched the tachycardia cycle length.
